# Air pollution by allergenic spores of the genus *Alternaria* in the air of central and eastern Europe

**DOI:** 10.1007/s11356-014-4070-6

**Published:** 2015-01-17

**Authors:** Idalia Kasprzyk, Victoria Rodinkova, Ingrida Šaulienė, Olga Ritenberga, Agnieszka Grinn-Gofron, Malgorzata Nowak, Aneta Sulborska, Joanna Kaczmarek, Elzbieta Weryszko-Chmielewska, Elena Bilous, Malgorzata Jedryczka

**Affiliations:** 1 Department of Environmental Biology, University of Rzeszow, Zelwerowicza 4, 35-601 Rzeszow, Poland; 2 Vinnitsa National Pirogov Memorial Medical University, Vinnitsa, Ukraine; 3 Department of Environmental Research, Siauliai University, Šiauliai, Lithuania; 4 Faculty of Geography and Earth Sciences, University of Latvia Riga, Riga, Latvia; 5 Department of Plant Taxonomy and Phytogeography, University of Szczecin, Szczecin, Poland; 6 Laboratory of Aeropalynology, Faculty of Biology and Department of Dermatology, Adam Mickiewicz University and University of Medical Sciences, Poznan, Poland; 7 Department of Botany, Lublin University of Life Sciences, Akademicka 13, 20-950 Lublin, Poland; 8 Institute of Plant Genetics, Polish Academy of Sciences, Strzeszynska 34, 60-479 Poznan, Poland

**Keywords:** *Alternaria*, Aerobiology, Spatial analysis, Meteorological parameters, Aeroallergen, Biological pollution

## Abstract

Spores of the genus *Alternaria* belong to one of the most prevailing constituents of the air in all regions of the world. They form infectious inoculum of numerous plant species as well as severe inhaled allergies. The aim of this study was to compare the biological pollution with *Alternaria* spores of the air of 12 cities located in central and eastern Europe. The experiment was done in 2010 and it covered the territory of Latvia (LV), Lithuania (LT), Poland (PL) and Ukraine (UA). The spores were counted using an identical method and standard equipment (7-day Lanzoni volumetric sampler) followed by extensive statistical calculations. The timing of the day of maximum concentration changed mainly along the N-S direction and had a positive correlation with latitude. The most important factor determining the increase in *Alternaria* spore concentration was the temperature, whereas other weather parameters were not related or of low significance. Regardless of geographical location, the first phase of the season (0–0.9 % of *Alternaria* spores in the air) was the longest (up to 60 days) and the last (97.5 to 99 %) was the shortest (22 days or less). The means of daily concentrations of *Alternaria* spores ranged from 11 spores m^−3^ in Klaipeda (LT, Baltic Sea coast) to 187 in Poznan (west PL, agricultural plain). The threshold value of 80 spores m^−3^ that triggers the first allergy symptoms was exceeded in 8 to 86 days (Vinnitsa, UA, temperate continental, forest-steppes region). There were considerable differences between the highest number of spores per cubic metre of air, varying from 139 in the north (Klaipeda, LT) to 2,295 in central west (Poznan, PL). The biological pollution by *Alternaria* spores in several places of central and eastern Europe was high; the number of days exceeding the threshold value of 300 spores m^−3^ connected with serious health problems of atopic people ranged from 0 to 1 on the north (LV, LT) to 29 in central west (Poznan, PL).

## Introduction

The genus *Alternaria* Nees ex Wallroth belongs to the phylum Ascomycota (www.mycobank.org). It comprises cosmopolitan dark-coloured fungi of the class Dothideomycetes occurring in all climatic zones. Members of the genus tolerate a wide range of temperatures, but the most abundant spore production and mycelium development are observed at 22 to 28 °C, while growth and development hardly occurs at sub 0 °C temperatures (Hjelmroos 1993). Under laboratory conditions, sporulation occurs at 8-24 °C and the conidia are fully mature within 14–24 h of their initiation (Mamgain et al. 2013). Based solely on morphology, Simmons (2008) divided the genus *Alternaria* into 276 species that were described in detail in an identification manual. Recently, Woudenberg et al. (2013) employed nucleotide sequence data from fragments of the 18S nuclear DNA, 28S nuclear DNA, internal transcribed spacer, glyceraldehyde 3-phosphate dehydrogenase, RNA polymerase II (RPB2) and translation elongation factor 1 alpha (TEF-1α) gene regions to redefine species identification within the genus. On this basis, the *Alternaria* species complex contains 24 internal clades, referred to as sections, and six monotypic lineages. Consequent upon this study, the genera *Allewia*, *Brachycladium*, *Chalastospora*, *Chmelia*, *Crivellia*, *Embellisia*, *Lewia*, *Nimbya*, *Sinomyces*, *Teretispora*, *Ulocladium*, *Undifilum* and *Ybotromyces* are proposed as synonyms of *Alternaria*, thus further widening the results of de Hoog and Horre (2002) that was based on ITS sequences only.

In most cases, species of *Alternaria* are either obligate or facultative pathogens; they may also grow as saprotrophs or endophytes and have been isolated from water and substrates such as soil, plants, organic matter, textiles, plaster or wood. Plant pathogenic species and saprophytes cause serious economic agricultural losses (Lou et al. 2013; Mamgain et al. 2013; Gerbore et al. 2014). Infected potatoes, oilseed rape, or cereals produce less tubers or seeds. Sporulation of the associated *Alternaria* species lowers crop quality and causes spoilage of agricultural products as well as food, during transport and storage (Humpherson-Jones 1989; Escuredo et al. 2011).

Species of *Alternaria* may also adversely affect human health. It has been estimated that 12 to 42 % of atopic people are mould sensitive (Knutsen et al. 2012) and about 70 % of patients respond to the presence of *Alternaria* spores in air samples (D’Amato and Spieksma 1995; Sanchez and Bush 2001). Although ten types of *Alternaria* allergens have been identified, the most damaging and frequently reported is a 31-kDa glycoprotein designated as Alt a1. The frequency of positive skin prick tests varied from country to country. Knutsen et al. (2012) reported 12.9 % positive skin prick tests among US citizens aged 6 to 59 years. Approximately 3 % of the Portuguese population suffered from allergy to either *Alternaria* or *Cladosporium*, while in Spain positive skin tests to both fungi were reported for 20 % of the population (Licorish et al. 1985; D’Amato et al. 1997). The intensity of allergic reaction of patients to the presence of *Alternaria* spores in air samples depends on the concentration of spores in the air. According to Rapiejko et al. (2004), the threshold concentration in Poland amounts to 80 spores m^−3^ of air, whereas Gravesen (1979) published a threshold value 20 spores higher. In sensitive patients, severe inhaled allergy symptoms are usually recorded at 300 *Alternaria* spores m^−3^, both in central Europe and elsewhere (Gravesen 1979; Black et al. 2000; Downs et al. 2001; Rapiejko et al. 2004)

Conidia of *Alternaria* were feature prominently in the long-duration fungal air spora of many European countries. Considerable differences have been observed in duration, dates and fluctuation of airborne fungal spores as well as the influence of geographic location, humidity, distance from the sea, climatic zone (latitude) and geobotanic conditions on their daily concentrations (Adams 1964; Nikkels et al. 1996; Peternel et al. 2004; Oliveira et al. 2009; Mikaliũnaité et al. 2009; Maya-Manzano et al. 2012; Sabariego et al. 2012). In Sweden and Denmark, the period of high aerial concentration of *Alternaria* spores is short. The periods of the highest concentrations were recorded in the first half of August and ended in mid-September (Hjelmroos 1993; Skjøth et al. 2012). In central and western Europe, spore concentrations were higher than those of North Europe, rising gradually from mid-April and remaining at a high or relatively high level until the end of September. The highest concentrations were usually recorded in July and August (Nikkels et al. 1996; Stepalska et al. 1999; Kasprzyk et al. 2004; Mikaliũnaité et al. 2009).

In Romania, the maximum concentrations of *Alternaria* spores in the air were found in May (Brasov, Timisoara) or July (Bucharest) (Ianovici and Dumbravă 2008). On the Iberian Peninsula, *Alternaria* spores were present in the air in significant concentrations throughout the whole year, even in the winter months (Maya-Manzano et al. 2012; Aira et al. 2013). In north-western Spain and Italy, the highest concentrations of conidia of *Alternaria* spp. were found between July and September (Rodriguez-Rajo et al. 2005; Rizzi-Longo et al. 2009). In the south of the Iberian Peninsula, the highest concentrations of spores were recorded in spring (April-June), late summer and early autumn (September-October) (Recio et al. 2012; Aira et al. 2013). In Turkey (Edrin), there were also two periods of high concentrations of airborne spores of *Alternaria* spp. The highest concentrations were found in June and July and then in September and October (Celenk et al. 2007).

Arable fields are considered as the main source of *Alternaria* spores; hence, the seasonal fluctuations and patterns are affected by the seasonal agronomic activities on farmlands (Corden et al. 2003; Skjøth et al. 2012; Aira et al. 2013). Climate is another factor that strongly influences the distribution of spores. Weather conditions affect the sporulation, transport and deposition of spores. Positive correlations between temperature and sunlight and negative correlations with relative humidity and rain have been reported (Grinn-Gofron and Rapiejko 2009; Oliveira et al. 2009; Escuredo et al. 2011; Sabariego et al. 2012). Based on a weather required for spore dispersal, the genus *Alternaria* was placed among fungal species that produce ‘dry spores’. In Stockholm (Sweden), a relative humidity above 45 %, winds and more than 60 % overcast skies reduced the concentration of *Alternaria* spores (Hjelmroos 1993). The maximum daily concentrations were usually at noon and in the afternoon, when the temperatures were at their highest and the humidity reached the lowest values (Hjelmroos 1993; Peternel et al. 2004; Recio et al. 2012).

As related above, a number of reports have described the occurrence of *Alternaria* spores in the air from different regions. However, in most cases, the monitoring was done at single site only (Hjelmroos 1993; Nikkels et al. 1996; Corden et al. 2003; Celenk et al. 2007; Skjøth et al. 2012). Significantly fewer papers focused on comparison between monitoring sites and within sites with similar climatic and agricultural conditions (Stepalska et al. 1999; Grinn-Gofron and Rapiejko 2009; Mikaliũnaité et al. 2009; Tomasetti et al. 2009). Most of studies have been conducted in Spain and Portugal (Munuera Giner et al. 2001; Rodriguez-Rajo et al. 2005; Oliveira et al. 2005, 2009; De Lineres et al. 2010; Maya-Manzano et al. 2012; Recio et al. 2012; Sabariego et al. 2012). Aira et al. (2013) carried out long-duration and detailed studies on almost the entire area of the Iberian Peninsula (12 stations) over regions that differ in bio-climatic conditions. The authors compared spatial and temporal differences between the presence of spores, the total annual spore counts and daily concentrations of *Alternaria* spores in the air. This study was made in the context of differences in climate and weather conditions. Hitherto, no comparable study has been carried out in central and eastern Europe, a vast region differing in climate, weather and flora. Our aim was to correct this oversight by comparing the dynamics of the occurrence of *Alternaria* spores in the air in selected sub-regions of this geoclimatic zone. We present the first set of data of this kind ever collected; no data from Latvia were available prior to this study, and data on monitoring in Lithuania and Ukraine were very scarce. Based on the assumption of vagaries in fluctuations in the occurrence of airborne *Alternaria* spores, their concentrations and timing of seasonal release, arising from the type of climate and geographical location, we embarked on testing the hypothesis that the nature of the relationship between meteorological elements and the concentration was similar over this region regardless of the site.

## Materials and methods

### Site location

**Fig. 1:**
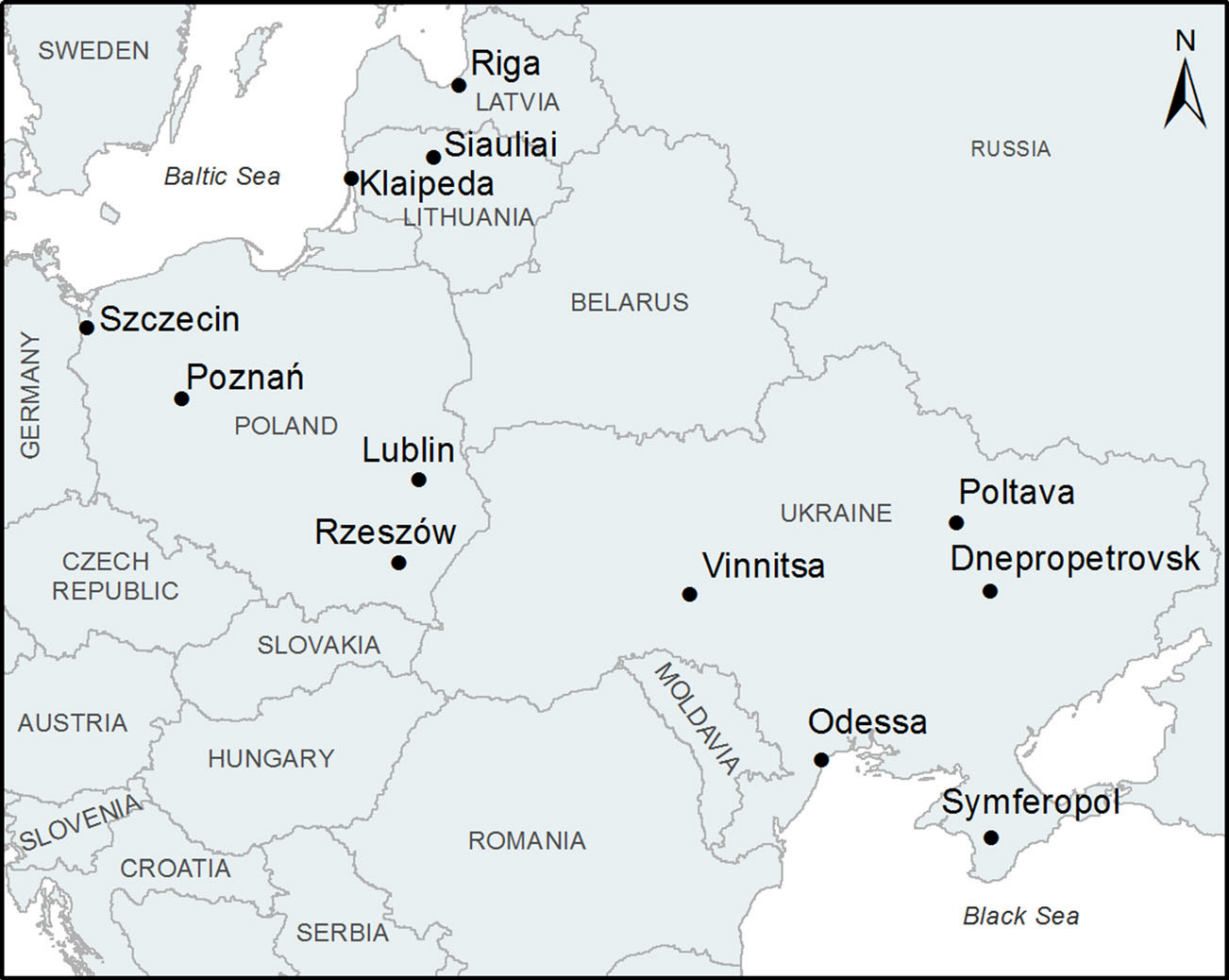
The location of 12 monitoring sites in central and eastern Europe used in this paper

The aerobiological monitoring was carried out in 12 cities located in central and west Europe (Fig. 1). Szczecin and Simferopol are the most geographically distant sites. On a NW-SE transect, the distance between them is above 2,000 km. Along transect N-S, the furthest situated sites are Riga and Simferopol and the distance between them is about 1,500 km. Dnepropetrovsk and Odessa are the biggest cities with the highest populations, close to 1 million citizens. The smallest city is Siauliai with around 120 thousand citizens.

The central and northern parts of the study area are located in lowland areas, whereas the southern sites were located in uplands. The region of study is in the temperate warm climate zone. Ukraine is characterized by continental warm type of climate whereas the rest have a transitional one (Martyn 2000). There are climatic differences connected with NW-SE maritime-continental gradient. The general trend is an increase in the continental index from the west of Poland toward central Ukraine. Proximity to seas modifies this tendency. Szczecin, Klaipeda and Riga are located on the coasts of the Baltic Sea, and Odessa and Simferopol on the Black Sea. The lowest mean annual rainfall and the highest mean annual temperature are in Odessa and Simferopol. The coldest city is Siauliai, where the mean annual temperature is more than 7 °C lower than in Simferopol (Table 1). In the last decade, the growing season in Szczecin lasted above 225 days, in Poznan, Rzeszow 225 and in Lublin 215 days (Nierobca et al. 2013). In Ukraine, the longest growing season, above 240 days, is at the Black Sea. Towards the north of the study area, the growing season is shorter; Dnepropetrovsk, 210 days; Riga, 185–190 days. Proximity to the Baltic Sea modifies this pattern. In eastern Lithuania, it is 170 days, while it is above 200 days in Klaipeda. Areas of Ukraine south of Poltava are located in the steppes zone. Deciduous and mixed forests dominate in western and northern regions of Ukraine and Poland. In Lithuania and Latvia, the most common vegetation are mixed forests with pine, spruce and birch.

**Table 1:** Basic information about aerobiological monitoring sites in central and eastern Europe used in this paper

Monitoring site	Country	Height of trap above ground level	Geographical coordinates	Height of site above see level	Type of climate	Type of landscape	Mean annual temp. [°C]	Mean annual sum of precipitation [mm]
Riga	Latvia	23	N 56° 95′ E 04° 11′	27	Temperate (humid-continental)	City centre	6.5	655
Siauliai	Lithuania	18	N 55° 55′ E 23° 18′	152	Middle latitudes of the temperate zone	City centre	6.0	568
Klaipeda		23	N 55° 75′ E 23° 12′	42	Marine	Suburb: pine-birch forest	7.0	735
Szczecin	Poland	21	N 53° 26′ E 14° 32′	60	Temperate with a clear influence of the sea	City centre	7.3	741
Poznań		18	N 52° 27′ E 16° 55′	102	Temperate with clear influence of oceanic climate	Suburban	8.5	500
Lublin		18	N 51° 14′ E 22° 32′	197	Temperate warm	City centre	8.6	638
Rzeszów		12	N 50° 01′ E 22° 00′	220	Temperate warm	City centre	8.8	734
Dnepropetrovsk	Ukraine	15	N 48° 46′ E 34° 98′	142	Temperate continental.	City centre	10.4	615
Odessa		22	N 46° 48′ E 30° 74′	49	Temperate continental, warm and dry	City centre	11.5	362
Poltava		15	N 49° 58′ E 34° 55′	181	Temperate continental	City centre near park area	9.6	606
Simferopol		18	N 44° 94′ E 34° 11′	181	Temperate continental with influence of the sea	City centre	12.7	322
Vinnitsa		25	N 49° 22′ E 28° 44′	258	Temperate continental	City centre	8.3	714

For most cities in this study, the aerobiological stations were located in the city centre. In Poznan, the spore trap was situated in a suburban area, and in Klaipeda, the monitoring station was surrounded by forests with conifers (Pinaceae) as dominant trees.

### Aerobiological monitoring

Aerobiological study was carried out from 1 April to 30 September in 2010. A volumetric method was used with Hirst-type spore traps (Hirst 1952) produced by Lanzoni (Bologna, Italy). A rotating drum inside each trap moved at a constant speed of 2 mm h^−1^ and attained one full rotation per week. It was covered by a Melinex tape coated with sticky fluid (silicone or Vaseline in toluene). Air (with spores) was sucked continuously into the trap at a rate of 10 L min^−1^. After full cycle, the Melinex tape, with trapped spores, was removed and cut into seven equal pieces corresponding to each day of sampling, followed by mounting on glass slides for light microscopic observation (Frenguelli 2003).

**Fig. 2:**
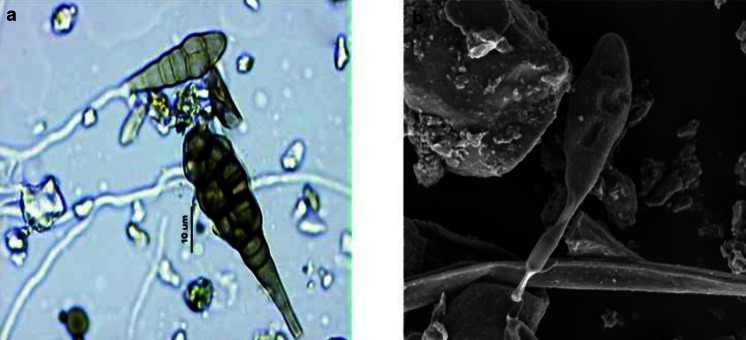
*Alternaria* spores: **a** light microscope; **b** scanning microscope (Phot. A. Sulborska)

*Alternaria* spores were identified on the basis of conidial shape, characteristic conidial septation and dark pigmentation (Fig. 2), and counted with a light microscope at ×400 magnification from one horizontal strip of a glass slide (48 mm long). The results were expressed as average daily concentration per cubic metre and the total spore count in whole season as Seasonal Fungal Index (SFI). Airborne spore occurrence was determined using the percentage method. The day on which the cumulative sum reached the value of 1 % of the seasonal total was considered to be the start of the season, whereas the day when the sum reached the value of 99 % was considered to be its end.

### Statistical analysis

Shapiro-Wilk test showed that the spore concentrations were not normally distributed and the Brown-Forsyth test demonstrated that variances were not homogenous. This is why nonparametric tests were used for further statistical analysis (Shapiro et al. 1968; Brown and Forsythe 1974). Null hypothesis about the lack of differences in the mean *Alternaria* spore concentrations between 12 cities was verified using single factor Kruskal-Wallis test followed by post-hoc Dunn’s test for multiple comparisons (Kruskal 1952). The strength and direction of correlations between the daily values of meteorological parameters and daily spore concentrations were assessed by Spearman correlation coefficients. To this analysis, several parameters were included: temperature (T, °C), relatively air humidity (H, %), precipitation (PP, mm), wind speed (WS, m/s). The relationships between coordinates and chosen season’s characteristics were tested using Pearson correlation as these data were normally distributed (Zar 1999). The statistical hypotheses were tested with *p* ≤ 0.05. In order to evaluate similarity between the cities in respect of some seasons’ features (total and monthly spores sums, maximum concentrations), the hierarchical clustering analysis was applied. This multidimensional technique enabled the determination of the groups (clusters) of cities with the greatest similarity.

## Results

The total seasonal number (SFI) of *Alternaria* spores varied significantly between cities. It ranged from 34,165 in Poznan to 1,956 in Klaipeda. In Riga and Siauliai, the values for SFI were low (between 3,000 and 4,000 spores). Similar trend was observed in the maximum concentration of *Alternaria* spores in air samples. In Poznan, the peak value exceeded 2,000 spores m^−3^, whereas in Klaipeda this value was only 139 spores m^-3^, i.e. 16 times less spores than in Poznan. Similar low peak value was noted in Riga (Table 2).

**Table 2:** Chosen descriptive statistics of airborne *Alternaria* spore seasons at 12 sites located in central and eastern Europe in 2010

City (country code)	Seasonal sum of fungal spores							Percent of days with spores	The highest no. of spores per cubic metre	Day of the highest no. of spores per cubic metre	Number of days exceeding threshold values			
	Apr-Sep	Apr	May	Jun	Jul	Aug	Sep				≥80 s/m^3^	>100 s/m^3^	>150 s/m^3^	>300 s/m^3^
Riga (LV)	3,286	13	48	67	731	2,358	69	61.7	206	10 August	13	8	4	0
Klaipeda (LT)	1,956	20	145	26	608	1,037	126	48.1	139	15 August	8	3	0	0
Siauliai (LT)	4,071	13	178	152	1,031	2,332	350	62.3	377	3 August	13	9	6	1
Szczecin (PL)	19,286	298	214	1,777	10,667	5,883	447	98.9	1300	11 July	56	48	34	22
Poznan (PL)	34,165	101	233	949	11,662	19,337	1,883	83.1	2295	2 August	58	53	44	29
Lublin (PL)	22,295	56	255	854	9,154	11,033	943	96.7	1615	19 June	66	2	32	23
Rzeszow (PL)	9,780	38	97	1,004	3,878	4,332	431	77.0	694	15 July	42	36	27	3
Dnepropetrovsk (UA)	15,729	76	312	1,362	6,038	2,809	5,129	84.7	575	17 July	61	53	36	15
Odessa (UA)	15,379	67	291	3,953	5,338	2,603	3,127	90.7	987	30 June	68	54	29	8
Poltava (UA)	21,630	144	1483	1,979	11,460	2,606	3,958	96.7	987	29 July	71	57	39	22
Simferopol (UA)	12,803	22	80	1,969	5,016	2,202	3,514	83.1	689	4 July	58	50	33	3
Vinnitsa (UA)	22,674	207	666	2,522	9,458	6,331	3,490	97.8	1,489	20 July	86	76	55	20

*LV* Latvia, *LT* Lithuania, *PL* Poland, *UA* Ukraine

**Fig. 3:**
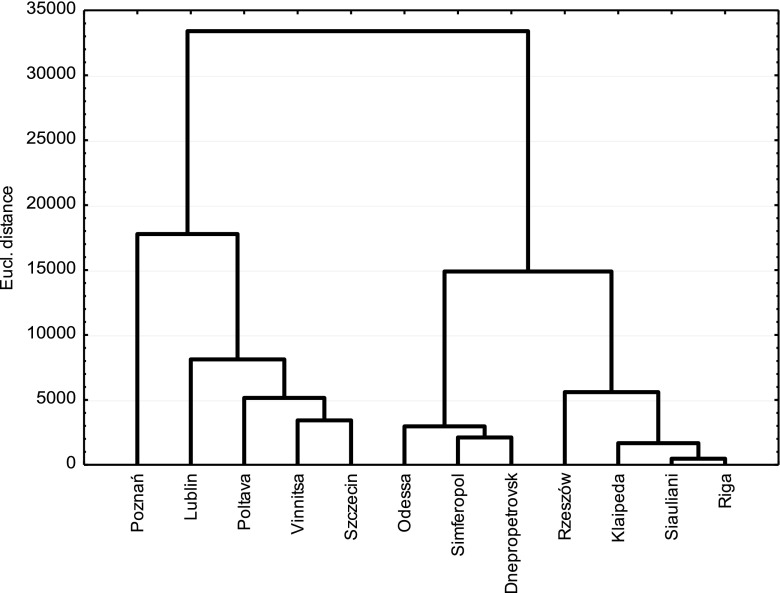
Dendrogram clustering sites according to the highest similarity of *Alternaria* spore concentrations in consecutive months and maximum spore concentration

Three groups of cities were distinguished judging by maximum concentration and monthly distribution of spore numbers. The first one was characterized by the highest value in August and the lowest value of the maximum concentration and SFI. This group comprised Riga (LV), Klaipeda (LT), Siauliai (LT) and Rzeszow (PL). The second variant consisted of cities located in the south (Odessa, Simferopol and Dnepropetrovsk, all located in Ukraine), where the highest spore counts were observed in July, with an intervening August, followed by a second peak in September. The seasonal concentrations were higher (ca. 13,000 to 16,000 of *Alternaria* spores). April was the month with the lowest concentration of spores in these two groups. The cities of the third group were distinct from those in the earlier groups in respect to much higher concentration of spores. Nevertheless, similarly to the first group, the highest spore counts were also observed in August (Table 2, Fig. 3).

The means of daily concentrations of *Alternaria* spores ranged from 11 spores m^−3^ in Klaipeda to 187 spores in Poznan. There were no differences in average daily spore counts between cities located in northern part of the area studied, i.e. Riga (Latvia) as well as Klaipeda and Siauliai, located in Lithuania (Table 3). In these cities, the number of days, when *Alternaria* spore concentrations exceeded the threshold value (80 spores m^−3^) that trigger allergy symptoms, ranged from 8 to 13. The highest number of days with concentrations above this threshold value was observed in Ukraine with 86 days in Vinnitsa, and 71 or 68 days in Poltava and Odessa, respectively (Table 2).

**Table 3:** Probability of errors in Kruskal-Wallis test for comparison of mean daily *Alternaria* spore concentration in the air of 12 monitoring sites across central and eastern Europe

City (country code^a^)	Riga	Klaipeda	Siauliai	Szczecin	Poznan	Lublin	Rzeszow	Dnepropetrovsk	Odessa	Poltava	Simferopol	Vinnitsa
Riga (LV)		NS	NS	0.000	0.000	0.000	0.000	0.000	0.000	0.000	0.000	0.000
Klaipeda (LT)	NS		NS	0.000	0.000	0.000	0.000	0.000	0.000	0.000	0.000	0.000
Siauliai (LT)	NS	NS		0.000	0.000	0.000	0.024	0.000	0.000	0.000	0.000	0.000
Szczecin (PL)	0.000	0.000	0.000		NS	NS	0.023	NS	NS	NS	NS	NS
Poznan (PL)	0.000	0.000	0.000	NS		NS	NS	NS	NS	NS	NS	0.046
Lublin (PL)	0.000	0.000	0.000	NS	NS		0.000	NS	NS	NS	0.023	NS
Rzeszow (PL)	0.000	0.000	0.024	0.023	NS	0.000		NS	0.000	0.000	NS	0.000
Dnepropetrovsk (UA)	0.000	0.000	0.000	NS	NS	NS	NS		NS	NS	NS	0.011
Odessa (UA)	0.000	0.000	0.000	NS	NS	NS	0.000	NS		NS	NS	NS
Poltava (UA)	0.000	0.000	0.000	NS	NS	NS	0.000	NS	NS		0.046	NS
Simferopol (UA)	0.000	0.000	0.000	NS	NS	0.023	NS	NS	NS	0.046		0.001
Vinnitsa (UA)	0.000	0.000	0.000	NS	0.046	NS	0.000	0.011	NS	NS	0.001	

*NS* no statistical significance

^a^Country code as in Table 2

**Fig. 4:**
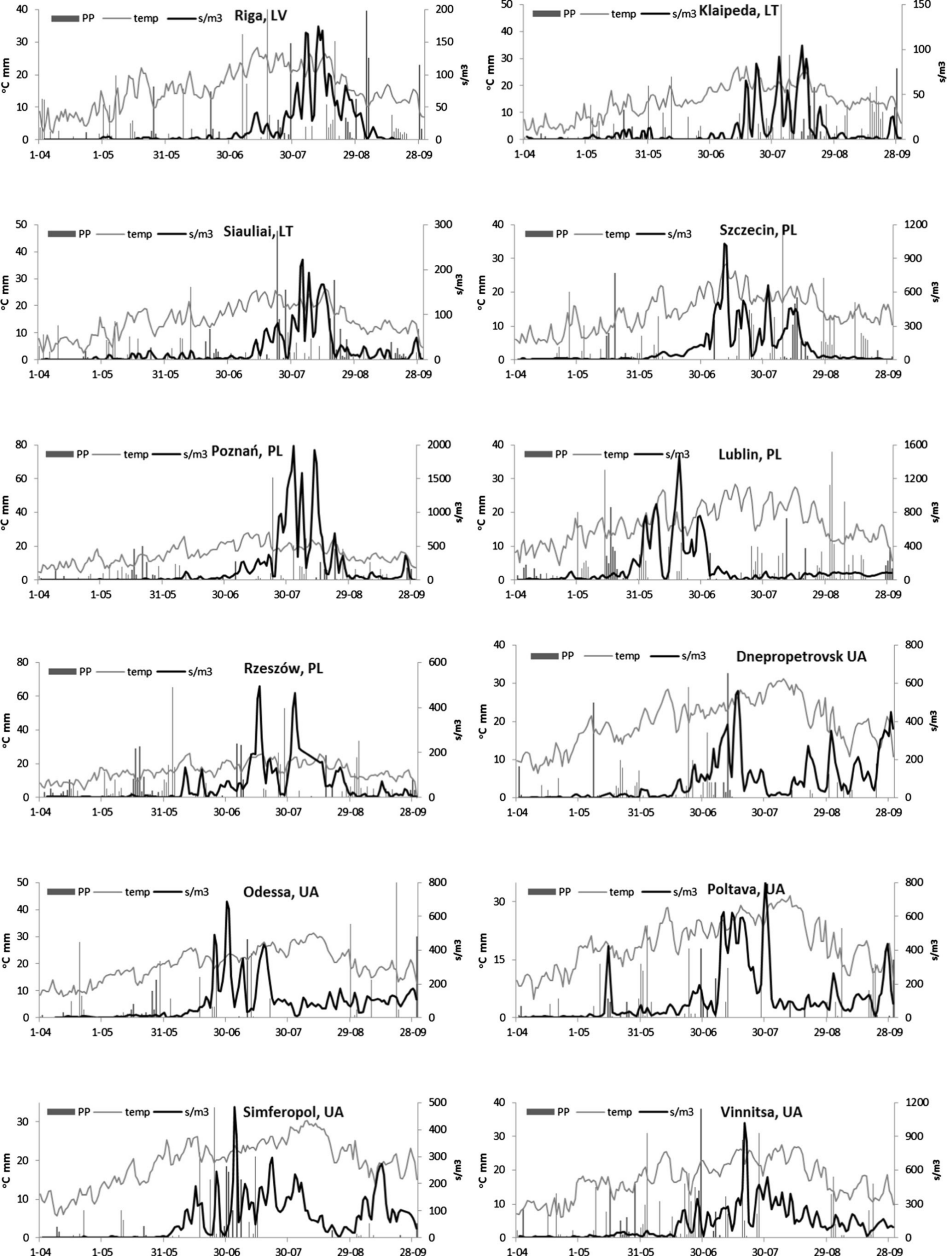
The patterns of rainfall (*PP*, *bars*), mean temperature (*grey line*) and daily concentrations of *Alternaria* spores (*black line*) in the air of 12 cities in central and eastern Europe in 2010 (*LV* — Latvia, *LT* — Lithuania, *PL* — Poland, *UA* — Ukraine)

The frequency of incidence of spores in the air differed among cities. In the more northerly cities, the frequency was the lowest, viz 48.1 % in Siauliai and 62.3 % in Klaipeda. There were several periods without airborne spores, especially in the pre-peak periods. In Szczecin, Lublin, Poltava and Vinnitsa, spores were recorded almost each day (Table 2). The seasons of this study had different patterns. With the exception of Riga, no clearly defined maximum was observed in any other city. In Riga, the concentrations increased in the second half of July, peaked in August followed by a short post-peak period. As in Vinnitsa, the seasonal pattern of incidence was relatively dense and roughly symmetrical. In Siauliai and Klaipeda, the seasonal fluctuations had similar patterns. In other cities, the seasonal patterns were less clearly defined as very long pre-peak periods with low spore numbers were often succeeded by rapid increases in concentrations. Such a scenario was observed in Szczecin, Poznan and Odessa. In Dnepropetrovsk, Poltava and Simferopol, the post-peak periods were very irregular (Fig. 4).

The seasons generally overlapped with each other. The correlation coefficients, which connote synchronicity, were relatively high. The similarities among the seasonal distributions were not connected with the closeness in geographical distance. Although Lublin is located near Rzeszow, synchrony of the seasons was not evident. The closest similarities were found amongst cities located farthest in the SE direction. The lowest, but statistically significant correlation was between Klaipeda and Odessa (*r* = 0.185) and the highest for Poznan and Vinnitsa (*r* = 0.752). Poznan overlapped, to a large degree, with seasonal patterns observed for Riga, Szczecin, Rzeszow and Simferopol; in all cases, the coefficients were above 0.7 (Table 4).

**Table 4:** Spearman correlation coefficients as a mean of the synchronisation of airborne *Alternaria* seasons between studied sites across central and eastern Europe

City (country code^a^)	Riga	Klaipeda	Siauliai	Szczecin	Poznan	Lublin	Rzeszow	Dnepropetrovsk	Odessa	Poltava	Simferopol	Vinnitsa
Riga (LV)		0.522	0.643	0.641	0.738	NS	0.630	0.489	0.402	0.534	0.506	0.682
Klaipeda (LT)	0.522		0.639	0.299	0.500	NS	0.445	0.294	0.185	0.380	0.320	0.407
Siauliai (LT)	0.643	0.639		0.489	0.626	NS	0.541	0.364	0.280	0.496	0.457	0.565
Szczecin (PL)	0.641	0.299	0.489		0.715	NS	0.743	0.446	0.458	0.508	0.595	0.646
Poznan (PL)	0.738	0.500	0.626	0.715		NS	0.723	0.582	0.523	0.626	0.702	0.752
Lublin (PL)	NS	NS	NS	NS	NS		NS	0.294	0.214	0.226	0.270	0.212
Rzeszow (PL)	0.630	0.445	0.541	0.743	0.723	NS		0.436	0.473	0.552	0.596	0.643
Dnepropetrovsk (UA)	0.489	0.294	0.364	0.446	0.582	0.294	0.436		0.727	0.726	0.649	0.649
Odessa (UA)	0.402	0.185	0.280	0.458	0.523	0.214	0.473	0.727		0.661	0.577	0.617
Poltava (UA)	0.534	0.380	0.496	0.508	0.626	0.226	0.552	0.726	0.661		0.693	0.694
Simferopol (UA)	0.506	0.320	0.457	0.595	0.702	0.270	0.596	0.649	0.577	0.693		0.703
Vinnitsa (UA)	0.682	0.407	0.565	0.646	0.752	0.212	0.643	0.649	0.617	0.694	0.703	

^a^Country code as in Table 2

**Fig. 5:**
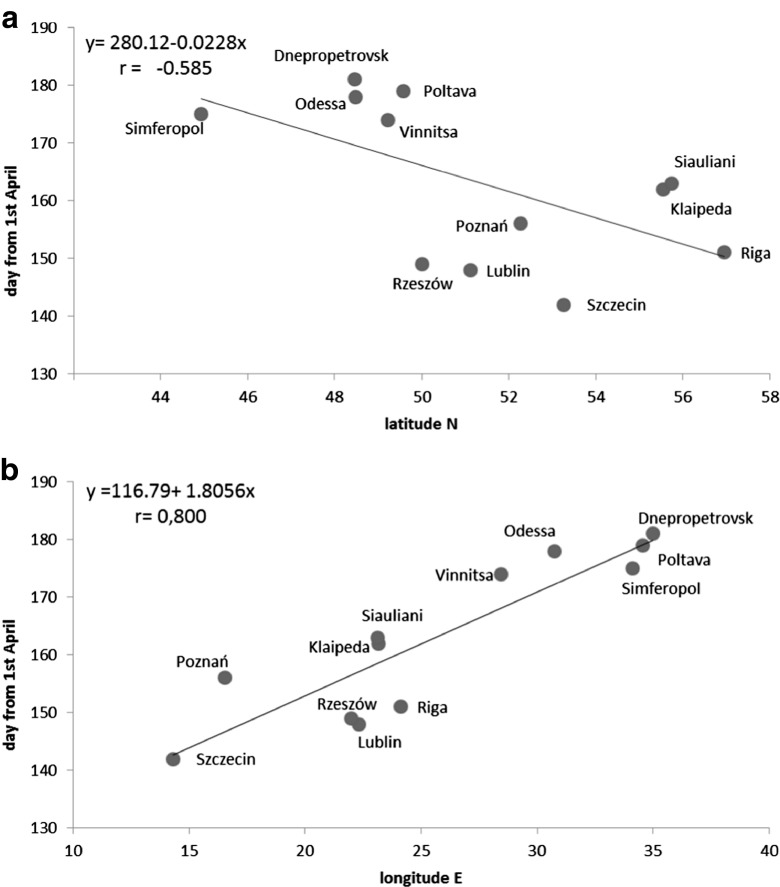
The correlation between the day of 90 % phase of *Alternaria* spore season and geographical location according to **a** latitude (N), **b** longitude (E)

**Fig. 6:**
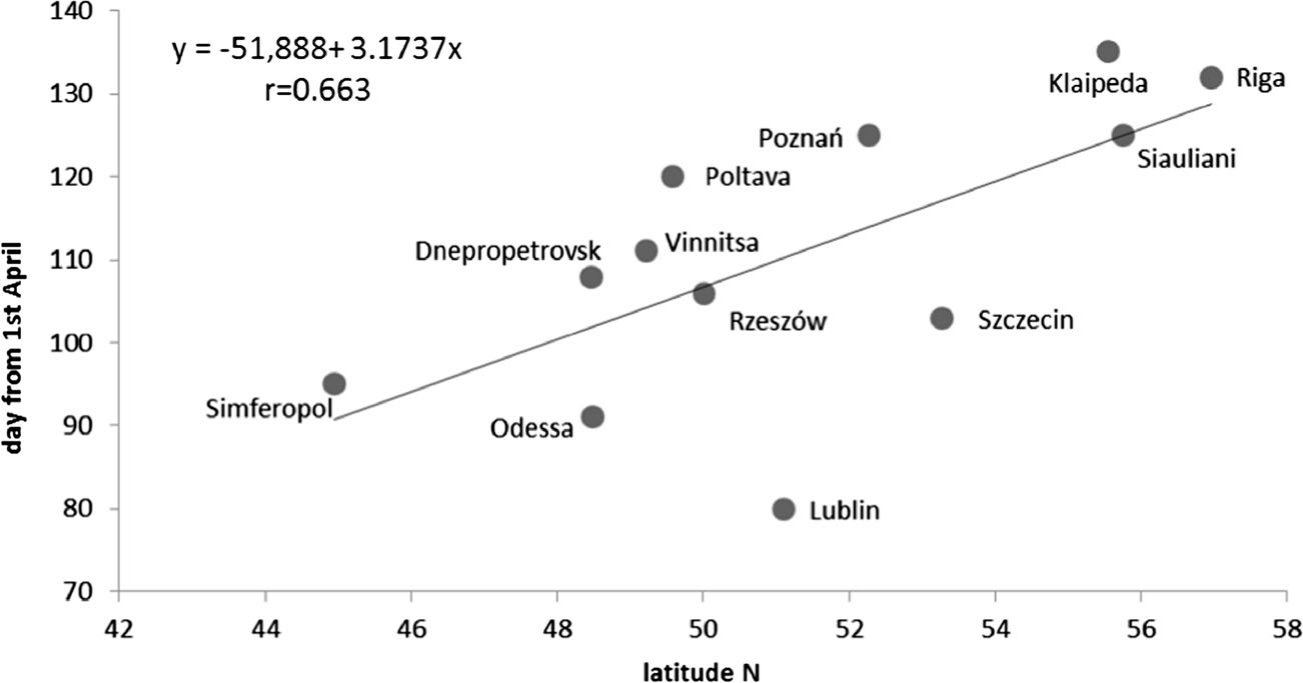
The correlation between the day of peak concentration of *Alternaria* spores and latitude (N) at 12 monitoring sites across central and eastern Europe

Some characteristics of *Alternaria* spore season depended on geographical location. In the area of study, the timing, when cumulative count of spore reached 90 %, was longer in the south-easterly direction. The correlation coefficient between that phase and latitude was *r* = 0.800. The correlation coefficient with the longitude was lower (*r* = −0.585) but also statistically significant. Towards SE direction, 90 % phase delayed (Fig. 5). The timing of the day of maximum concentration changed mainly along the N-S direction and had a positive correlation with latitude. In Lublin, the date of maximum airborne spore concentration was the earliest observed in the current study. Cities located continentally in the south namely, Dnepropetrovsk, Vinnitsa, Poltava and Rzeszow attained maximum concentrations of airborne *Alternaria* spores later. Northern cities such as Riga and Klaipeda attained maximum concentrations on the latest (Table 2, Fig. 6). However, correlations could not be established between the start of other phases of seasonal fluctuations in air spora and geographical location.

Temperature was the most important factor determining the increase in *Alternaria* spore concentrations in all sites of the current study, except Lublin. The strength of this relationship differed significantly. The strongest positive correlations were found for temperature in Szczecin (mean temperature, *r* = 0.795) and Poznan (minimum temperature, *r* = 0.795). In Odessa and Dnepropetrovsk, the values of correlation coefficients were the lowest (maximum temperature, *r* = 0.442; mean temperature, *r* = 0.376). In Rzeszow, however, other factors besides temperature also influenced airborne *Alternaria* spore counts, as weak but statistically negative significant correlations were obtained for humidity, precipitation and wind speed (*r* = −0.290; *r* = −0.404; *r* = −0.232). Rzeszow was the only city where rainfall was observed to affect spore concentration. In Szczecin and Vinnitsa, wind speed and maximum wind speed slightly determined the decrease of spore concentrations. Among all the cities of this study, data from Lublin were exceptional in that none of the meteorological parameters appeared to have affected airborne spore counts (Table 5).

**Table 5:** Spearman correlation coefficient for *Alternaria* spore concentrations in the air of 12 sites across central and eastern Europe and selected meteorological parameters (April-September 2010)

City (country code)	Tmean	Tmax	Tmin	H	PP	WS	Max WS
Riga	0.705	0.643	0.729	NS	NS	NS	NS
Klaipeda	0.566	0.569	0.537	NS	NS	−0.152	–
Siauliai	0.644	0.612	0.631	NS	NS	−0.264	–
Szczecin	0.795^a^	0.782^a^	0.640	−0.369^a^	NS	−0.280^a^	−0.209^a^
Poznań	0.749	0.675	0.795^a^	NS	NS	NS	NS
Lublin	NS	NS	NS	NS	NS	NS	NS
Rzeszów	0.710	0.697	0.626	−0.290	−0.404^a^	−0.232	NS
Dnepropetrovsk	0.376	0.389	0.379	NS	NS	NS	NS
Odessa	0.448	0.442	0.446	NS	NS	NS	NS
Poltava	0.611	*0.622*	0.582	NS	NS	NS	NS
Simferopol	0.611	0.575	*0.643*	NS	NS	–	–
Vinnitsa	0.565	0.551	*0.584*	0.161	NS	−0.199	−0.146

Items in italics are the highest coefficients for a given site

*T* temperature, *H* relative air humidity, *PP* precipitation, *WS* wind speed

^a^The highest coefficients for a given meteorological parameter

## Discussion

In 2010, the concentration of *Alternaria* spores over the territory of Poland did not differ significantly from those of the previous years; the fluctuations and the maximal values were within the range of variation reported in earlier studies (Kasprzyk et al. 2004; Stepalska and Wolek 2005; Kasprzyk and Konopinska 2006; Mikaliũnaité et al. 2009). It is noteworthy that the highest concentration of the spores of *Alternaria* was again observed in Poznan (Great Poland region), situated in central west of the country. It was in line with the results obtained in 1995–1996, when a comparative study was conducted in several regions of Poland. However, big differences in spore concentrations were found between the results obtained in the current study and those of earlier years. In 1995, the highest concentration of *Alternaria* spores exceeded 8,000 spores m^−3^ and rose the following year to over 24,000 spores m^−3^. However, in 2010, the maximum concentration of *Alternaria* spores in Poznan was several times lower.

Aerobiological characteristics of the season, especially the timing and maximum values for Rzeszow, located in south east Poland, close to the border with Ukraine were in a similar range of variation as they were in 2000–2002 (Kasprzyk et al. 2004; Kasprzyk and Worek 2006). In 2010 in Rzeszow however, the sum of the maximum concentration of *Alternaria* spores was significantly lower as compared to Lublin, located 150 km up north. Comparable results were previously reported in 2002 (Kasprzyk and Konopinska 2006). In 2010 in Szczecin, located in the very north-west part of Poland, the highest concentration of *Alternaria* spores was found in July, similarly to the observations done in 2006 (Grinn-Gofron and Rapiejko 2009). In 2004–2005, very high concentrations were also recorded in August. Generally, the seasonal sums of spores and the other parameters recorded in 2010 were in the range of previously observed variation.

In the case of Vinnitsa (Ukraine), the concentration of *Alternaria* spores in 2010 was much higher than in 2009, which can be explained by higher temperatures in 2010 (data not shown). Hence, in both years, the situations were totally different from each other, showing two extremes. In Lithuania (Klaipeda and Siauliai), the fluctuation of *Alternaria* conidiospores in 2010 was similar to 2005–2006, but concentrations in 2010 were lower than described in previous reports (Mikaliũnaité et al. 2009), which contrasted with the opposite trend in Ukraine.

Average daily concentration of airborne spores is a highly dynamic parameter. In the area covered by the current study, there were great differences between the mean daily concentrations of spores. No significant differences in average daily concentrations of spores were observed in the cities located in the north east of the region, particularly in Riga, located in Latvia and two Lithuanian cities: Klaipeda and Siauliai. Similarly, over the period of a 10-year study in Stockholm, the highest daily average concentration of *Alternaria* did not exceed 25 spores m^−3^ and peak concentrations ranged between 50 and 70 spores m^−3^ (Hjelmroos 1993). The other studied locations in central and eastern Europe differed greatly from each other. The highest numbers of spores varied from around 600–700 spores m^−3^ in cities located both in Ukraine (Dnepropetrovsk, Simferopol) and south-east Poland (Rzeszow) to as high as 1,600–2,000 spores in central and north-west Poland (Poznan and Szczecin, respectively). Regional differences were reported for airborne *Alternaria* spores in Romania; however, the values ranged from 10 to 92 spores m^−3^ and maximum values of 30 to 600 spores m^−3^ of the air (Ianovici and Dumbravă 2008). Differences of maximum spore concentrations of *Alternaria* presented in this paper are big, however not as dramatic as reported by Stepalska et al. in 1995–1996 (1999).

Regional fluctuations of spore concentrations of *Alternaria* depend on land use and the type of landscape (Corden et al. 2003; Awad 2005; Mikaliũnaité et al. 2009; Maya-Manzano et al. 2012; Sabariego et al. 2012). In Denmark, as well as in Poland, high spore concentrations decreased when the leaves with sporulating cultures of the fungus abscised. Skjøth et al. (2012) proposed agricultural lands as the main sources of these spores, since they are the habitat for numerous host plants of this pathogenic genus and our study suggest this hypothesis is justified. Poznan, where annual total concentrations of *Alternaria* spores were exceptionally high, is the capital of Great Poland—a region of large hectarages of cereals, oilseed rape, potatoes and other arable crops. These monoculture fields play important roles in the development of fungal species belonging to *Alternaria*. In southern Poland, in the region of Rzeszow, there are numerous forests instead and less agriculture, with smaller farms and diversified crops. This may be the main reason for lower concentration of *Alternaria* spores, in comparison with typical arable agricultural areas of west Poland.

Agronomic treatments may greatly affect the spore concentration detected in the air samples. During the field crop management (e.g. at harvest), the concentration of *Alternaria* might rise to levels that are critical for people with allergies. Since *Alternaria* spp. are pathogenic to agricultural crops such as cereals and oilseed rape, increased spore concentration would occur at harvest, provided the weather conditions were favourable for sporulation, spore release and dispersal of these pathogens (Corden et al. 2003; Skjøth et al. 2012; Aira et al. 2013). In India, sudden build-up of spore concentration coincides with the harvest of rice. In Turkey, this phenomenon coincides with haymaking as well as harvest (Chakrabotry et al. 2003; Celenk et al. 2007). In Denmark, the main sources of *Alternaria* spores within Copenhagen were local or regional areas, but long distance transport was also listed among possibilities (Skjøth et al. 2012). Harvest operations in central Europe (Sadys et al. 2014) can influence episodes of high concentration of spores in Denmark, so the forecasts should take into account the possibility of long transport.

A number of workers have emphasized the influence of seasonal variability of meteorological conditions (particularly temperature, rainfall and relative humidity) on the dynamics and seasonal fluctuations of airborne fungal spores including those of *Alternaria* spp. (Sakiyan and Inceoglu 2003; Sabariego et al. 2012). Our results confirm that *Alternaria* belongs to fungal genera producing ‘dry spores’, for which temperature is the most important factor, determining increases in daily spore concentrations in air samples. Based on literature data, the role of precipitation, relative humidity and wind on the presence and concentration of *Alternaria* spores is not clear. In the current study, elements of weather other than temperature either had no effect on daily spore concentration, or only very weak influence was found. The patterns of concentrations of *Alternaria* spores observed in 2010 in air samples from all selected cities of Latvia, Lithuania and Ukraine as well as three out of four cities in Poland were not associated with the amounts of rain or relative air humidity. Only in Rzeszow (south-east Poland), a weak relationship between these parameters was demonstrated.

In many fungal species spore germination, host infection, mycelial growth and sporulation are promoted by dew or high air relative humidity (Peternel et al. 2004; Celenk et al. 2007; Aira et al. 2013). However, the case might not be so for spore dispersal (Timmer et al. 1998; Peternel et al. 2004). The concentration of *Alternaria* spores in Poland was sometimes strongly correlated with temperature and low air humidity (Stepalska and Wolek 2005; Grinn-Gofron and Strzelczak 2008). Rodriguez-Rajo et al. (2005) reported a negative correlation between length and severity of rainfall and concentration of spores. Prolonged rain washed spores from the air over long duration (Hjelmroos 1993; Maya-Manzano et al. 2012). In Szczecin (north-west Poland), a significant increase of *Alternaria* spore concentration was observed during the period of higher air temperature and ozone concentration prior to a thunderstorm (Grinn-Gofron and Strzelczak 2013). Rodriguez-Rajo et al. (2005) found that the optimal temperature for high airborne *Alternaria* spore concentration ranged between 23 and 29 °C, in combination with relative humidity values of about 80 %. In Turkey, increase in *Alternaria* spore concentration was observed in June, when air temperature rose and the relative humidity was increased by frequent showers (Celenk et al. 2007). In Stockholm (Sweden), concentrations of airborne *Alternaria* spores were sensitive to variation in air humidity and cloudiness. It was noted that *Alternaria* spore concentration was higher, when the relative humidity increased above 45 %, total cloudiness was over 60 % and the winds were rather strong (Hjelmroos 1993).

The current results confirm the trend that in Europe, the period of high concentration of *Alternaria* spores is shorter in the more northerly locations (Hjelmroos 1993; Nikkels et al. 1996; Oliveira et al. 2009; Rizzi-Longo et al. 2009). In this study, in regions located in north-east Europe, the period of the highest concentrations of *Alternaria* spores was the shortest, with peak days in August. The lowest spore concentration was observed in April. Such a pattern coincided with annual fluctuations in air temperature. In this region, springs are cooler than autumn, and for cities located in bays (such as Riga and Klaipeda), August is often the warmest month of the year (Martyn 2000).

Changes in spore concentration in Poznan (central-west Poland) and Rzeszow (south-east Poland) did not differ from those reported by earlier workers (Stepalska et al. 1999; Kasprzyk et al. 2004) and they were typical of western Europe, where the highest concentrations of *Alternaria* spores often occur in August (Nikkels et al. 1996). The study of Aira et al. (2013) performed at the Iberian Peninsula demonstrated that climate may be one of the factors influencing the fluctuations of spore concentrations within each season. In coastal cities, and in the centre of the peninsula, *Alternaria* spores were trapped continuously from spring to autumn, and the highest concentrations were observed in the summer months. In the continental climate of the south of Spain (Sevilla, Merida, Malaga), seasonal fluctuations were mostly bimodal; the highest concentrations were recorded in July and also — after a few weeks break — in October. The trough between these peaks might be due to very warm masses of tropical air, leading to physiological drought of plants (Martyn 2000). In Toledo, in central Spain, also characterized by continental climate, a bimodal season was also observed. However, the first peak occurred in June and the second — usually lower — was found in September (Sabariego et al. 2012). In the current study, in the continental area of south Ukraine (Odessa, Simferopol), the seasonal pattern of spore distribution was also bimodal. The highest monthly sums of *Alternaria* spores were recorded in July and — following a decline in August — a smaller increase in spore concentrations was again observed in September. In August, owing to continental air masses, these areas usually experience drought (Martyn 2000). In this study, similarities were observed in the dynamics of spores captured in similar types of climate.

Results obtained by Aira et al. (2013) at the Iberian Peninsula suggest that not only the season but also the dynamics of seasonal or annual sum of spores of *Alternaria* may be related to the distance from the ocean. In the north western regions and areas close to the Atlantic Ocean, spore concentrations were lowest and the season was shorter than in the south of Spain. Lower concentrations of *Alternaria* spores in coastal regions were also found in England (Corden et al. 2003; Morrow Brown and Jackson 1978). In the current study, the lowest sums of *Alternaria* concentrations, ranging from *ca*. 2,000 to 4,000 spores m^−3^, were found in Latvia and Lithuania, situated in north-east Europe, on the Baltic Sea coastline. Monitoring sites in this region had the lowest average annual temperatures among the locations studied. In contrast, in Szczecin (north-west Poland), also situated close to the Baltic Sea, concentrations of *Alternaria* spores were among the highest. These observations suggest that air temperature is a crucial parameter, most probably via an indirect effect on the length of plant vegetation period. In north-west Poland, the average annual temperature is higher than in Latvia or Lithuania and growing seasons are longer. It is reasonable to speculate that these variables influence, affect and determine the low values of annual total numbers of *Alternaria* spores in north Europe. A similar phenomenon was observed in Stockholm, where cumulative concentrations of fungal spores ranged from 1,000 to about 2,000 (Hjelmroos 1993). Moreover, comparable correlation was observed also in respect to fungal spores of other phytopathogens, belonging to *Leptosphaeria maculans*-*Leptosphaeria biglobosa* species complex (Kaczmarek et al. 2014). The studies undertaken in parallel in Lithuania and Poland also showed significantly less spores and shorter spore seasons in the north (Kedainiai district, Lithuania), as compared to experiment site located inland, in south-east Poland (Piliponyte-Dzikiene et al. 2014). This example also supports the hypothesis of the primary importance of temperature on *Alternaria* spore production and release, over rainfall and relative humidity.

In this study, Poznan was found to be a city with exceptionally high seasonal concentrations of *Alternaria*, reaching over 3,000 to 4,000 spores m^−3^ between April and September 2010. This result is in agreement with previous reports also indicating Poznan as a site with high concentration of *Alternaria* in air spora (Stepalska et al. 1999; Corden et al. 2003; Oliveira et al. 2009; Sabariego et al. 2012). In 1997, in Merida and Sevilla, located in central Spain, high annual total concentrations of *Alternaria* spores (over 50,000 and 40,000 spores m^−3^, respectively), and in 1998 again in Sevilla (over 30,000 spores m^−3^), were found (Aira et al. 2013). Similar observations were also reported for Leiden in the Netherlands in 1989. In the other years, the spore concentrations were significantly lower (Nikkels et al. 1996). The highest spore concentrations of *Alternaria* were recorded in Ankara (Turkey) in 1990, when monthly values summed up to almost 60,000 conidia within a single season (Sakiyan and Inceoglu 2003). Such high concentrations of spores were not detected in this study, even in the south of the monitored zone. However, daily concentrations of *Alternaria* exceeding 300 spores m^−3^— which is regarded as the threshold value for the most vexing health effects — were detected up to nearly 30 days in the season. This situation has a strong effect on human health, as in most cases such high daily spore concentrations of *Alternaria* cause severe asthma and the sensitive patients require professional ambulatory treatments.
